# Realtime cardiac function using compressed sensing: initial clinical evaluation

**DOI:** 10.1186/1532-429X-18-S1-Q1

**Published:** 2016-01-27

**Authors:** Jan Paul, Vanessa Schmitt, Stefan Wundrak, Michael Radermacher, Vinzens Hombach, Peter Bernhardt, Wolfgang Rottbauer, Volker Rasche

**Affiliations:** grid.410712.1Internal Medicine II, University Hospital of Ulm, Ulm, Germany

## Background

In current clinical routine, functional heart parameters are derived from breathhold CINE MRI. To enable free-breathing acquisitions and imaging e.g. in highly arrhythmic patients, realtime imaging is gaining interest. The increasing clinical availability of this new technique demands evaluation of functional parameters compared to the gold standard [1].

## Methods

### Study population and acquisitions

14 patients with different cardiac pathologies were randomly selected. All patients underwent our clinical routine imaging protocol and additionally real-time (RT) imaging in three short axis slices (apical, medial, basal). RT imaging was performed with a radial Tiny Golden Angle (Ψ_7_≍23.628°) [2] balanced acquisitions (α=60°) at 1.5T (Philips Achieva 1.5T).

### Reconstruction

Reconstruction from the retrospective gated CINE data (REF) were compared to compressed sensing realtime reconstruction via TyGRASP [3] (CS). Reconstructions were performed with a spatial resolution of 1.4 × 1.4 × 8 mm^3^ and temporal resolution of about 60 ms (CS) and 30 ms (REF).

### Analysis

ESV, EDV, SV, and EF were determined for LV and RV in the different reconstructions from the slices CS and matching slices of REF. Functional parameters were compared via correlation (Pearson, p-values below 5% were considered significant) and Bland-Altman analysis. Further analysis was performed by pairwise t-tests (α=5%, with Bonferroni correction for multiple testing).

## Results

**Figure 1:**
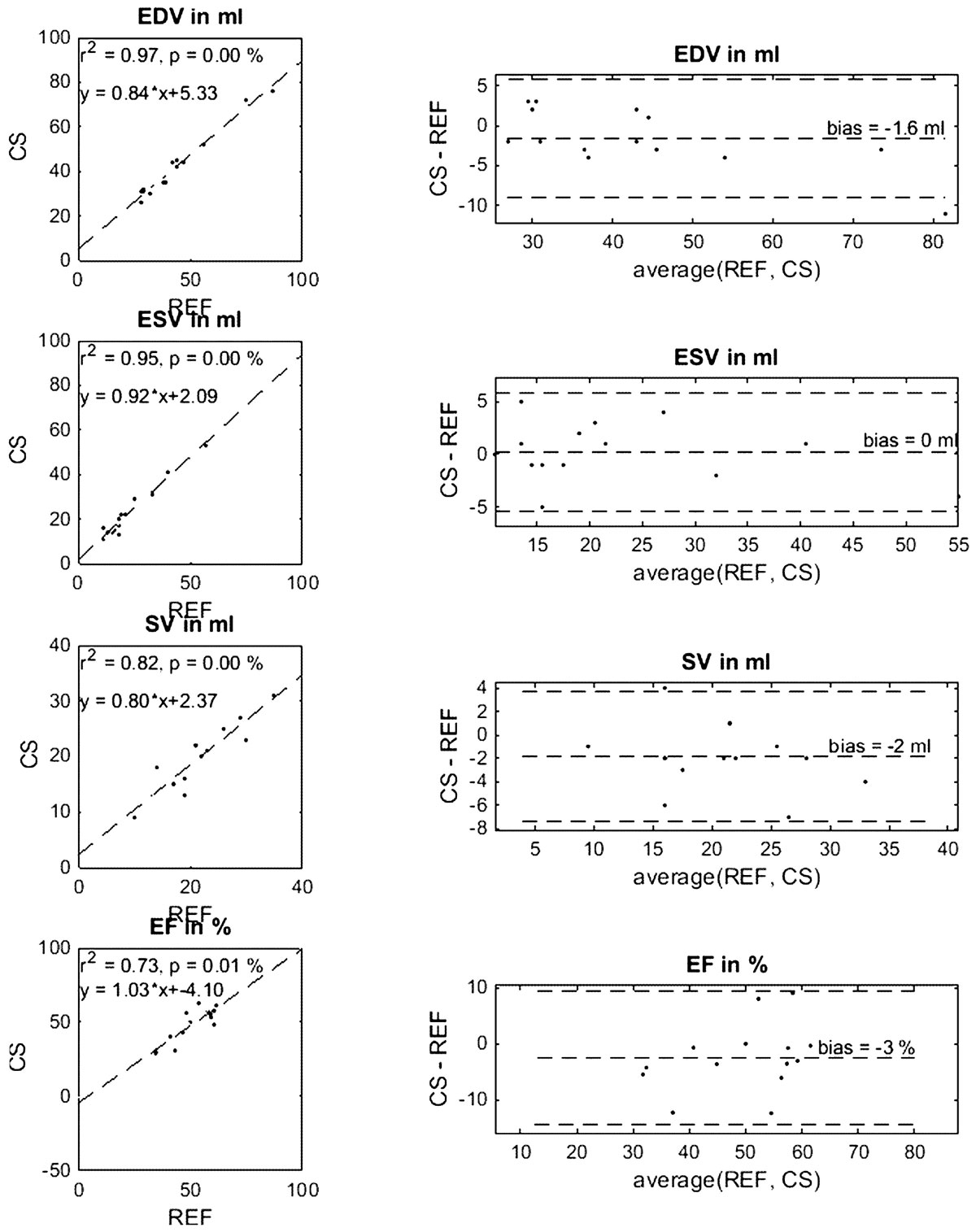
**Correlation and Bland-Altmant-Plots for comparison of End-Diastolic Volume (EDV), End-Systolic Volume (ESV), Stroke Volume (SV), and Ejection Fraction (EF) in the Left Ventricle (LV) for compressed sensing realtime (CS) compared to CINE (REF) reconstruction**.

**Figure 2:**
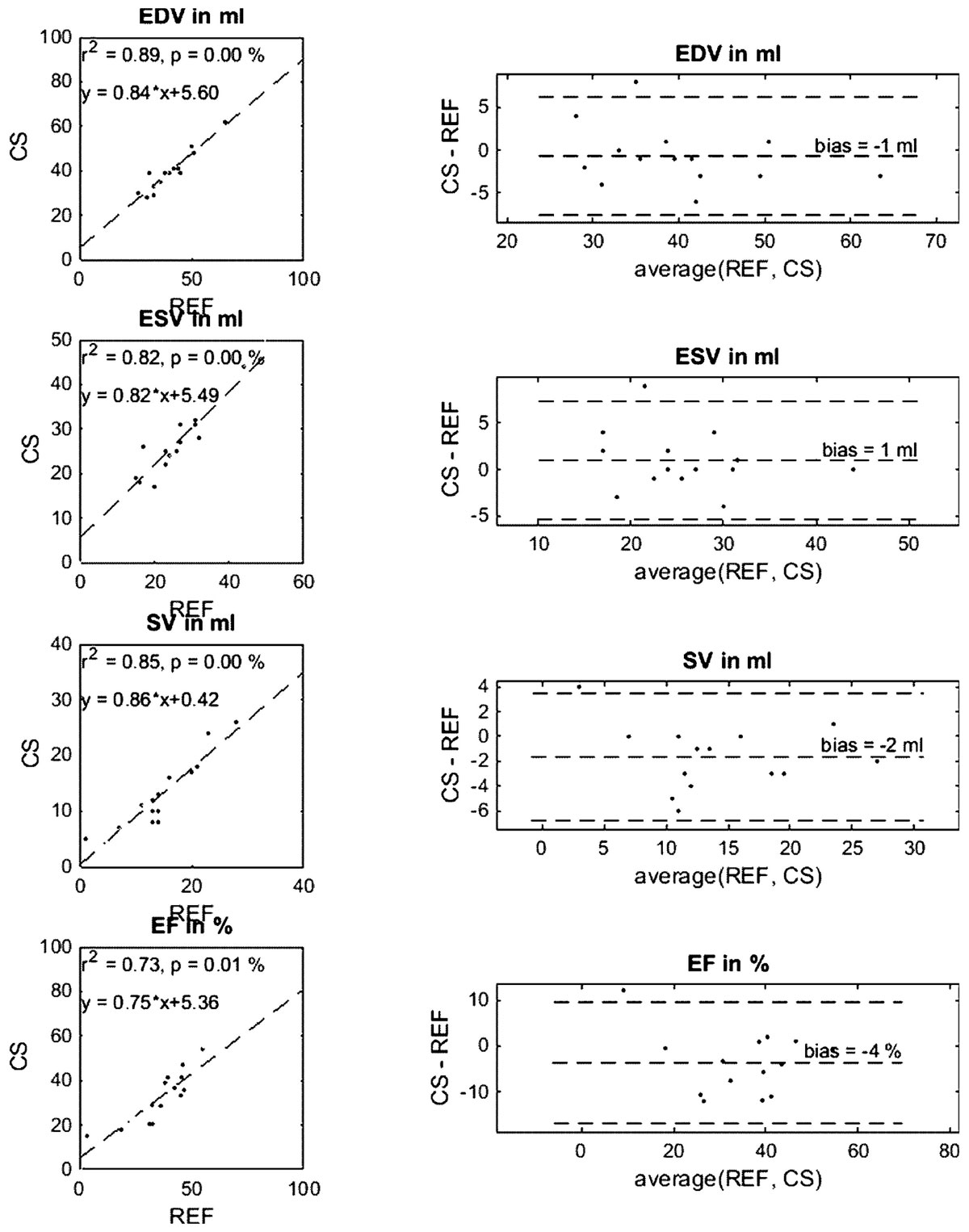
**Correlation and Bland-Altmant-Plots for comparison of End-Diastolic Volume (EDV), End-Systolic Volume (ESV), Stroke Volume (SV), and Ejection Fraction (EF) in the Right Ventricle (RV) for compressed sensing realtime (CS) compared to CINE (REF) reconstruction**.

Figures 1 and 2 show correlation and Bland-Altman-Plots for LV and RV, respectively. Good quality of fit (r^2^ > 0.7; all p < 5%) can be appreciated for all correlations. Only small average biases with slight underestimation of parameters in CS compared to REF are present both for LV and RV. T-tests showed no significant difference between parameters determined from CS or REF.

## Conclusions

The findings are in line with [1], where no significant bias was found for EF from realtime GRAPPA vs CINE in LV. The results suggest that the realtime technique is a promising for evaluation of cardiac function parameters. Further investigation is needed with more patients, full slice coverage and to evaluate the realtime technique during free-breathing. Additionally, sliding window realtime reconstruction without additional compressed sensing constraints might be investigated.
